# Dynamic Ensemble Learning with Transfer Learning for Fatigue Performance Prediction in Ni-Based Superalloys

**DOI:** 10.3390/ma19112371

**Published:** 2026-06-02

**Authors:** Jiaxing Yang, Fenglou Du, Haopeng Lv, Wang Li, Dayong Wu

**Affiliations:** Hebei Short Process Steelmaking Technology Innovation Center, School of Materials Science and Engineering, Hebei University of Science and Technology, Shijiazhuang 050018, China; yjx18230191050@126.com (J.Y.); 15637468529@163.com (F.D.); lwang199110@163.com (W.L.)

**Keywords:** Ni-based superalloys, feature alignment transfer learning, fatigue performance prediction

## Abstract

Accurate prediction of fatigue performance in Ni-based superalloys is hindered by scarce data and poor generalization of conventional machine learning. This study proposes a framework combining dynamic ensemble learning with transfer learning. A tensile prediction model using five base regressors (SVR, RFR, DTR, XGB, MLP) on 1025 tensile samples is first built. A dynamic weighted error feedback ensemble algorithm (DWELA) adjusts base model weights in real-time based on validation errors, improving tensile R^2^ from 0.90 (best single model) to 0.95. To transfer knowledge to fatigue prediction, a feature alignment transfer learning (FATL) strategy aligns shared features (composition and heat treatment) between source (tensile) and target (fatigue) domains while fine-tuning domain-specific strain features, adapting effectively to a limited fatigue dataset of 622 samples. The resulting ETFPM model evaluated on five independent samples achieves R^2^ of 0.93 (fatigue stress) and 0.81 (fatigue life), outperforming the best fatigue-trained single model (SVR: R^2^ = 0.89 and 0.72). Twenty candidate alloys are predicted for screening. The method offers a practical route for fatigue prediction under data-limited conditions. The main novelties are: (i) DWELA’s real-time error-driven weight adaptation with hard constraints and early stopping, which improves tensile R^2^ from 0.90 (best single model) to 0.95; and (ii) FATL’s explicit separation of frozen shared features and trainable exclusive features, enabling accurate fatigue prediction (R^2^ = 0.93 for FS, 0.81 for FL) using only 622 fatigue samples. However, the independent validation is limited to five samples, and the datasets are compiled from the literature with potential heterogeneity in testing protocols and imputation bias for missing values. Further experimental validation is required to confirm broader applicability.

## 1. Introduction

In aero engines and gas turbines, Ni-based superalloys [1] are critical for turbine blades and combustors thanks to their high-temperature strength, corrosion resistance, and stability. These components endure multi-field coupling (temperature, stress, fluid), and fatigue fracture is the primary failure mode. Accurate fatigue prediction is essential for safety and efficiency, and escalating performance demands urgently require efficient prediction methods [2].

Traditional R&D of Ni-based superalloys follows a sequential paradigm of “composition-smelting-process-testing”, which suffers from long cycles, high costs, and trial-and-error. Fatigue testing is especially challenging: a single low cycle fatigue data point costs an order of magnitude more than a tensile test, causing severe data imbalance (1025 tensile vs. 622 fatigue records in our database) [3]. Single machine learning algorithms lack generalization. Fixed weight ensembles cannot handle local heterogeneity, and conventional transfer learning lacks explicit feature alignment. While ensemble learning can integrate the advantages of multiple algorithms, current ensemble strategies predominantly use fixed weights, making it difficult to adapt to the dynamic nature of complex data. Transfer learning can leverage mature knowledge from source domains to address data scarcity in target domains, but traditional transfer methods lack precise adaptation to feature differences between source and target domains. Specifically, the existing literature lacks an ensemble method that (i) updates base model weights in real-time based on validation set errors, (ii) imposes hard constraints to maintain diversity, and (iii) uses early stopping based on ensemble error convergence all tailored for material property prediction with small datasets. Furthermore, conventional transfer learning either fine-tunes all layers (risking overfitting) or performs adversarial domain adaptation (requiring large target sets). No prior work has explicitly separated shared and exclusive features with frozen shared weights for tensile to fatigue knowledge transfer in Ni-based superalloys. Furthermore, insufficient training on small-scale fatigue datasets further constrains prediction accuracy. To address these gaps, this work exploits the metallurgical commonality between tensile and fatigue behavior, enabling knowledge transfer via explicit shared feature alignment. The scientific justification for this transfer lies in the fact that both yield strength (YS) and fatigue stress (FS) are governed by resistance to dislocation motion, which is controlled by the same underlying solid solution strengthening (Cr, Fe) and *γ*′ precipitation hardening (Al, Ti) mechanisms modulated by heat treatment [4]. Specifically, the early stage of fatigue damage is dominated by dislocation slip and planar slip band formation, which are directly hindered by solid solution atoms (Cr, Fe in the *γ* matrix) and coherent *γ*′ precipitates (Ni_3_(Al,Ti)). The same strengthening mechanisms control tensile yield strength, establishing a quantitative physical basis for knowledge transfer. Moreover, the Coffin–Manson relationship links plastic strain amplitude to fatigue life through dislocation substructure evolution, and the parameters of this relationship are known to depend on composition and heat treatment, further justifying the transfer of tensile learned features.

This study focuses on the precise prediction of tensile and fatigue performance in Ni-based superalloys. The core innovations include: (1) designing a dynamic weighted error feedback integration algorithm (DWELA), which dynamically adjusts weights based on real-time prediction errors to overcome the limitations of fixed weight integration [5]; (2) proposing a feature aligned transfer learning (FATL) strategy that achieves efficient transfer of integration models to fatigue prediction tasks through shared feature alignment and fine-tuned exclusive features [6]; and (3) establishing a complete framework of “basic prediction integration optimization transfer adaptation independent validation,” using five independent new datasets for final model validation to ensure generalization capability [7]. The research findings provide significant theoretical and engineering value for shortening alloy development cycles, reducing experimental costs, and ensuring safe service of high-end equipment.

## 2. Methods

### 2.1. Dataset Collection

Tensile Performance Dataset (TPD): All data were compiled from the single comprehensive public database accompanying the published work “*Data-driven prediction of fatigue performance in nickel-based superalloys*” [8]. From this source, 1025 valid samples were integrated, covering 30 nickel-based wrought high-temperature alloy grades. The inclusion criteria for a sample were: (i) complete records for at least the five core alloying elements (Ni, Cr, Fe, Al, Ti) and the four target tensile properties; (ii) heat treatment parameters falling within ranges typical for wrought Ni-based superalloys; and (iii) test temperature ≥ 20 °C. Each sample contains 12 input features (5 core chemical components: Ni, Cr, Fe, Al, Ti; 7 process and test parameters: solid solution temperature ST, solid solution time STt, stabilization aging temperature STat, stabilization aging time Stat, aging temperature AT, aging time At, test temperature T), and 4 output performance indicators (ultimate tensile strength UTS, yield strength YS, elongation at break EL, reduction of area RA). Chemical composition units are mass percentages (wt.%); process parameters and test temperatures are hours (h) and degrees Celsius (°C), respectively; and performance indicators are in MPa or percentages (%). Fatigue Performance Training Dataset (FPD-Train): Constructed by screening 622 low cycle fatigue samples from the “*Data-driven prediction of fatigue performance in nickel-based superalloys*”, covering the same alloy system’s composition, process, test conditions, and fatigue performance data. The fatigue dataset contains a total of 16 input features (5 core chemical components: Ni, Cr, Fe, Al, Ti; 6 process parameters: ST, STt, STat, Stat, AT, At; 4 test parameters: total strain range Δ*ε*_t_, elastic strain range Δ*ε*_e_, plastic strain range Δ*ε*_p_, test temperature T), and 2 output performance indicators (fatigue stress FS, fatigue life FL). The Fatigue Performance Independent Validation Dataset (FPD-Test) comprises 5 sets of new chemical component data that were not used in any training process, serving as an independent validation set to ultimately assess the generalization capability of the ETFPM model. These 5 samples were randomly selected from the same database, stratified by Ni content, and were completely excluded from all training, hyperparameter tuning, and normalization parameter calculation, thereby constituting a true out-of-sample test set. We use the model to predict output indicators and make a comparison with actual test values for validation [9].

Tensile tests followed ASTM E8/E21 (20–1200 °C, 10^−4^–10^−2^ s^−1^) [10,11]. Fatigue tests used fully reversed strain control (*R* = −1), strain amplitude 0.25–4.07%, temperature 20–1000 °C, frequency 0.1–1 Hz, with failure defined as a 50% load drop. Both datasets come from the same curated database, ensuring consistency.

All three datasets undergo strict preprocessing to meet model training and prediction requirements. Outliers were removed using the 3σ rule (12 out of 1025 tensile samples) [12]. Samples with >40% missing (8 samples) were discarded; for the remaining 23 samples with ≤40% missing, median imputation was used for compositions and properties, and process parameters were supplemented from the *China Superalloy Handbook* [13]. Finally, Z-score normalization was used to eliminate dimensional differences, converting all input and output features into standardized data with a mean of 0 and a standard deviation of 1, thereby removing dimensional influences between chemical compositions (wt.%), process parameters (h/°C), and performance indicators (MPa/%) to ensure the fairness and effectiveness of model training [14]. The normalization parameters (mean and standard deviation) were calculated exclusively from the training set and subsequently applied to the validation and test sets to prevent information leakage. FPD-Test preprocessing followed the same procedure. Supplementary Table S1 lists input/output ranges for tensile data; Supplementary Table S2 for fatigue data.

It is important to acknowledge the potential limitations arising from data origin and preprocessing. Both tensile and fatigue datasets are compiled from multiple literature sources, which may introduce unquantified variance due to differences in testing equipment, environmental conditions, and operator procedures. Although we applied outlier removal (3*σ* rule) and median imputation for missing values (≤40%), imputation bias remains possible, especially for process parameters supplemented from handbooks. The median filling approach may reduce the true variance of the data. Moreover, the applicability domain of the proposed model is restricted to wrought Ni-based superalloys within the composition and processing ranges listed in Supplementary Tables S1 and S2; extrapolation to cast alloys or other alloy systems requires dedicated validation. Readers should interpret the model predictions as screening suggestions rather than definitive replacements for experimental testing. Throughout the manuscript, units are consistently formatted: wt.% for composition, °C for temperature, h for time, MPa for stress, and cycles for fatigue life. Strain parameters are denoted as Δ*ε*_t_, Δ*ε*_e_, Δ*ε*_p_.

### 2.2. Construction of Basic Tensile Performance Prediction Model

A forward prediction model for tensile properties (TPFM) was systematically constructed using five classical machine learning [15] algorithm Support Vector Regression (SVR), Random Forest Regression (RFR), Decision Tree Regression (DTR), Extreme Gradient Boosting Regression (XGB), and Multilayer Perceptron Regression (MLP), which are well suited for material property prediction [16]. The model uses the 12 input and 4 output features described in Section 2.1. Four separate single output regression models were trained independently for UTS, YS, EL, and RA, rather than employing a multi-output regressor, due to distinct nonlinear relationships between inputs and each tensile property. During model construction, the feature distribution patterns of 1025 tensile datasets were fully adapted, with particular emphasis on capturing nonlinear correlations between core chemical components (Ni, Cr) and tensile properties [17]. This provides a stable and reliable foundational source model for subsequent dynamic integration and optimization algorithms. Initial parameters are set reasonably to balance training efficiency and fitting accuracy and ensure consistency with shared features in transfer learning [18]. This lays a solid foundation for the entire nickel-based superalloy performance prediction system.

Five algorithms were selected for their complementary strengths: SVR handles high-dimensional spaces [19]; RFR reduces overfitting via bootstrapping [20]; DTR provides interpretable splits [21]; XGBoost models complex interactions [22]; and MLP captures deep nonlinearity [23]. Hyperparameters were optimized via grid search with 5-fold CV: SVR (RBF, *C* = 10, *γ* = 0.1); RFR (200 trees, max depth = 15); DTR (max depth = 10, min samples split = 5); XGB (150 estimators, *lr* = 0.05, max depth = 6); MLP (hidden layers 64,32, ReLU, *lr* = 0.001).

The dataset is split into training, validation, and test sets by the industry standard. The 1025 tensile samples are split into 55%, 25%, and 20% to ensure consistent sample distribution and avoid evaluation bias from uneven data allocation [24]. This ratio was selected to ensure sufficient data for model training while maintaining a validation set large enough for stable weight computation in the DWELA ensemble algorithm. Five-fold cross-validation was employed during hyperparameter tuning; this was standard (non-nested) cross-validation, as the independent test set was strictly held out for final evaluation. Three common evaluation metrics for material property prediction are used: R^2^, RMSE, MAPE. In summary, TPFM uses 12 inputs, 4 separate single output models (SVR, RFR, DTR, XGB, MLP), grid search with 5-fold CV, 55/25/20 split, and R^2^/RMSE/MAPE metrics. They fully assess the model’s fitting quality, prediction error, and relative error. All models were implemented in Python 3.9.18 using scikit learn 1.3.0 (SVR, RFR, DTR, MLP) and XGBoost 2.0.2. Computations were performed on a workstation with an Intel Core i9-13900K CPU (64 GB RAM) and an NVIDIA RTX 4090 GPU (24 GB VRAM).

(1) $$R^{2}=1-\frac{\sum_{i=1}^{n}(y_{m\mathrm{eas}}-y_{p\mathrm{red}}{)}^{2}}{\sum_{i=1}^{n}(y_{m\mathrm{eas}}-{\bar{y}}_{m\mathrm{eas}}{)}^{2}}$$

(2) $$\mathrm{RMSE}=\sqrt{\frac{\sum_{i=1}^{n}(y_{\mathrm{pred}}-y_{\mathrm{meas}}{)}^{2}}{n}}$$

In the calculation formulas, *y*_meas_ denotes the actual value, *y*_pred_ the predicted value, and *ȳ*_meas_ the mean of actual values, with n representing the sample size. RMSE for fatigue life is reported in cycles; RMSE for stress and strength is reported in MPa.

### 2.3. Design of an Integrated Algorithm with Dynamic Weight and Error Feedback

The Dynamic Weighted Error Feedback Integration Algorithm (Hereafter referred to as DWELA) is proposed to solve single algorithm and fixed weight limits. DWELA adjusts weight coefficients in real time by checking each base algorithm’s prediction errors on the validation set. It takes 5 core components and process parameters as input and uses different algorithms’ advantages to capture component process performance correlations. It continuously improves tensile performance prediction accuracy. DWELA’s core advantage is its real-time error feedback and dynamic weight adaptation. It adjusts base algorithms’ weight proportions according to different input feature combinations’ prediction errors [25]. It avoids fixed weight problems such as poor adaptation to complex data distribution and no response to feature changes [26]. It also reduces the negative impact of high-error algorithms and maximizes strong-fitting algorithm advantages. DWELA differs from fixed averaging by real-time error feedback, from stacking by avoiding a meta learner, from boosting by parallel training, and from Bayesian averaging by deterministic weight updates. The fundamental novelty of DWELA over existing ensemble methods in materials informatics is threefold: (1) error-driven dynamic weight initialization based on validation RMSE rather than heuristic or uniform weights; (2) per-iteration weight adaptation using the ratio of previous to current errors, which directly responds to local performance changes; and (3) hard weight constraints [0.01, 0.6] combined with early stopping, which prevents any single model from dominating while maintaining diversity. In contrast, typical ensemble methods (e.g., random forest, XGBoost) use fixed or monotonic weights, and stacking relies on a meta learner that can overfit on small data. DWELA’s design is specifically optimized for scarce material datasets (1025 tensile samples) where dynamic re-weighting provides measurable gains (average R^2^ improvement from 0.90 to 0.95).

First, each base algorithm’s RMSEₖ on the validation set is calculated to quantify errors (*k* = 1, 2,…, 5 for the five base algorithms). Then, initial weights are assigned by the reciprocal of error, and algorithms with smaller errors get higher weights with a specific calculation formula [27].

(3) $$w_{k,0}=\frac{1/\mathrm{RMS}E_{k}}{\sum_{k=1}^{5}(1/\mathrm{RMS}E_{k})}$$

After each iterative training round, each algorithm’s prediction errors are updated to adjust weight coefficients dynamically. Weight is reduced if the error increases and increased if the error decreases with specific dynamic adjustment formulas.

(4) $$w_{k,t}=w_{k,t-1}\text{⋅}\frac{\mathrm{RMS}E_{k,t-1}}{\mathrm{RMS}E_{k,t}}$$

(5) $$w_{k,t}=\frac{w_{k,t}}{\sum_{k=1}^{5}w_{k,t}}$$

The ensemble model’s final predicted value is the weighted sum of each base algorithm’s predicted values with a specific calculation formula. Weights are updated using validation set errors only; an independent test set remains unseen throughout training and weight adjustment to prevent overfitting.

(6) $$y_{\mathrm{ensemble}}=\sum_{k=1}^{5}w_{k,t}\text{⋅}y_{k,\mathrm{pred}}$$

The complete DWELA procedure is summarized in Supplementary Algorithm S1. Initial weights are set inversely proportional to validation RMSE (Equation (3)). At each iteration, weights are updated by the error ratio (Equation (4)), normalized (Equation (5)), clipped to [0.01, 0.6], and re-normalized. The ensemble prediction is the weighted sum (Equation (6)). Early stopping triggers after five consecutive iterations with ensemble RMSE improvement < 10^−4^.

### 2.4. Feature Alignment Transfer Learning Strategy

**Figure 1:**
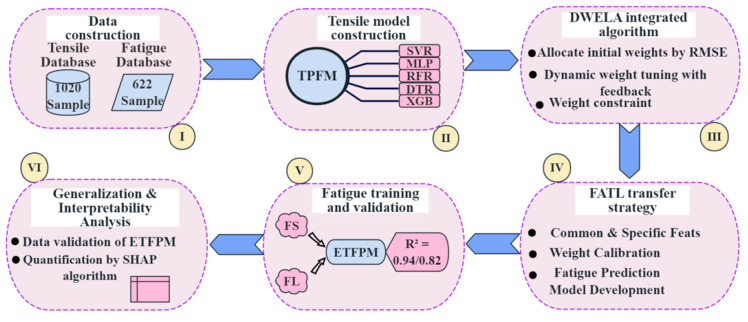
Flowchart of multi-model fusion and transfer learning for fatigue prediction. (**I**) Database construction; (**II**) TPFM with SVR, RFR, DTR, XGB, MLP; (**III**) DWELA (RMSE based initial weights, dynamic adjustment, constraints, early stopping); (**IV**) FATL (feature alignment); (**V**) ETFPM training; (**VI**) SHAP analysis.

**Figure 2:**
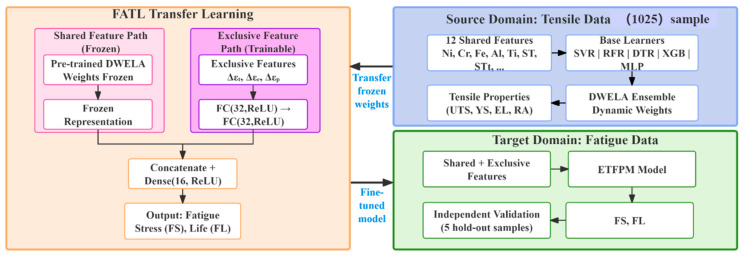
Architecture of the FATL transfer learning framework. The source domain (tensile data) pre-trains DWELA on 12 shared features. Shared features (composition and heat treatment) are frozen, while exclusive strain features (Δ*ε*_t_, Δ*ε*_e_, Δ*ε*_p_) pass through trainable branches (FC(32, ReLU) → FC(32, ReLU)). Concatenated features are fed into Dense(16, ReLU) to output fatigue stress (FS) and fatigue life (FL). The model is fine-tuned on the target domain (fatigue data) and validated on five independent samples.

Feature Alignment Transfer Learning (Hereafter, FATL) is proposed to apply the optimized DWELA model to fatigue prediction effectively. The complete procedure of the proposed method is summarized in Figure 1. FATL solves the feature distribution difference between the source domain (TPD) and target domain (FPD-Train) and takes five core chemical elements as shared features [28]. Feature alignment here refers to retaining the source trained weights for shared input features while adding new trainable branches for target specific features, without explicit distribution matching or adversarial training. The physical rationale for this transfer lies in the shared microstructural origin of tensile and fatigue properties: both are governed by solid solution strengthening (Cr, Fe) and *γ*′ precipitation (Al, Ti) modulated by heat treatment. During cyclic loading, fatigue crack initiation typically occurs at persistent slip bands or at interfaces between *γ* matrix and *γ*′ precipitates. The resistance to crack initiation is directly proportional to the yield strength, because higher strength reduces the cyclic plastic zone size and delays the formation of irreversible slip bands. Therefore, a model that accurately predicts yield strength from composition and heat treatment inherently captures the microstructural factors controlling fatigue crack nucleation. The exclusive strain features (Δ*ε*_t_, Δ*ε*_e_, Δ*ε*_p_) then modulate this crack initiation resistance according to the applied strain amplitude, reflecting the transition from crack initiation to propagation governed by the Coffin–Manson law. The architecture of the FATL transfer learning framework is illustrated in Figure 2. Shared features (5 elements, 6 process parameters, test temperature) are frozen; exclusive strain features (Δ*ε*_t_, Δ*ε*_e_, Δ*ε*_p_) are processed via new trainable branches and concatenated with the frozen representation [29,30]. The model is fine-tuned and trained on the FPD-Train dataset and cross-validation is used to optimize fine-tuning parameters [31]. Weights for the 12 shared features from the pre-trained DWELA model are transferred and frozen during fine-tuning. Weights for the three exclusive strain features are randomly initialized and trained on the FPD-Train dataset. Unlike conventional fine-tuning that updates all weights (which risks overfitting on small fatigue data) or adversarial domain adaptation that requires large target sets, FATL selectively retains and freezes shared feature weights while learning exclusive features via separate branches. The key differentiation of FATL from standard transfer learning approaches is: (1) explicit feature-type separation based on physical knowledge (composition/heat treatment as shared, strain parameters as exclusive); (2) complete freezing of the shared feature subnetwork, preserving tensile learned strengthening mechanisms; and (3) branch-specific training of exclusive features with random initialization, allowing independent learning of strain life relationships. This design is particularly advantageous when the target domain has very limited data (622 samples) and shared features have strong physical overlap with the source domain. This minimizes source and target domain feature distribution differences to combine source model knowledge and target domain features effectively. Aligned shared feature weights and fine-tuned exclusive feature weights are finally combined to build the Integrated Transfer Fatigue Performance Prediction Model (ETFPM) [32]. For FATL, shared feature weights from DWELA are retained, and three new branches for exclusive strain features (Δ*ε*_t_, Δ*ε*_e_, Δ*ε*_p_) are added and trained on FPD-Train using Adam (*lr* = 0.001, batch size = 32) for up to 100 epochs. Five-fold cross-validation with early stopping (patience = 10) is used to select the optimal epoch. It can predict fatigue stress and fracture life accurately and outputs 20 fatigue performance datasets. Its generalization ability is tested with 5 independent FPD-Test datasets to ensure reliable engineering application.

### 2.5. Visualization Analysis of Model Prediction Results

**Figure 3:**
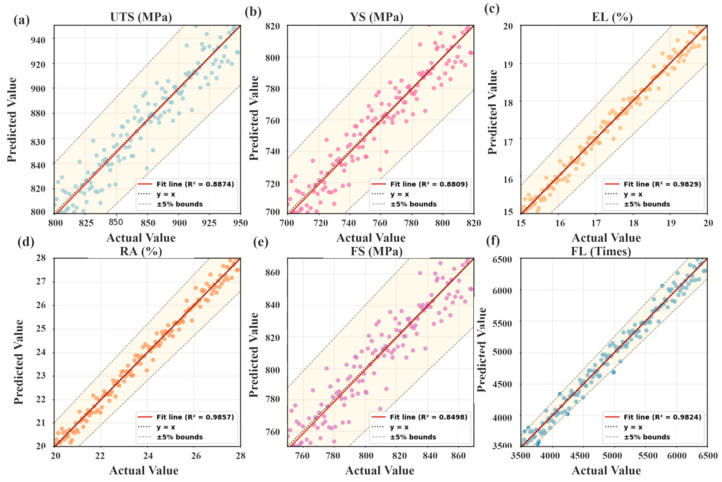
Model predictions for training and test datasets: (**a**) model predictions for tensile strength (UTS); (**b**) model predictions for yield strength (YS); (**c**) model predictions for elongation at break (EL); (**d**) model predictions for reduction at break (RA); (**e**) model predictions for fatigue strength (FS); (**f**) model predictions for fatigue life (FL).

Figure 3 shows the comparison between the optimal algorithm model and the DWELA algorithm model for predicting tensile properties (UTS, TYS, EL, RA, FS, FL). Scatter-point clustering along the diagonal directly reflects the match between model predictions and actual values.

The visualization shows the six tensile performance metrics cluster tightly along the diagonal with almost no dispersion. Tensile strength (UTS) and yield strength (YS) cluster the most closely. This matches the quantitative results exactly: the DWELA integrated model has an average R^2^ of 0.95 for tensile performance prediction and the R^2^ values for UTS and YS reach 0.95 and 0.94, respectively.

### 2.6. Model Interpretability Analysis

The SHapley Additive exPlanations (SHAP) algorithm is introduced to conduct interpretability analysis [33]. Based on the game theory principle of SHAP values, it fairly quantifies the contribution of each input feature to the prediction results, SHAP values were computed using KernelExplainer (SHAP v0.44.1), with 100 background samples randomly drawn from the respective training sets, addressing the ‘black box’ issue in traditional machine learning models [34]. This study looks at how five core chemical components work. SHAP values of features are calculated to make clear their positive or negative impacts and weight proportions on prediction results. Visualization tools like bee swarm graphs and feature dependency graphs are used to show clearly how core components relate to prediction results. SHAP analyses were performed separately for the DWELA model (tensile properties) and the ETFPM model (fatigue properties).

**Figure 4:**
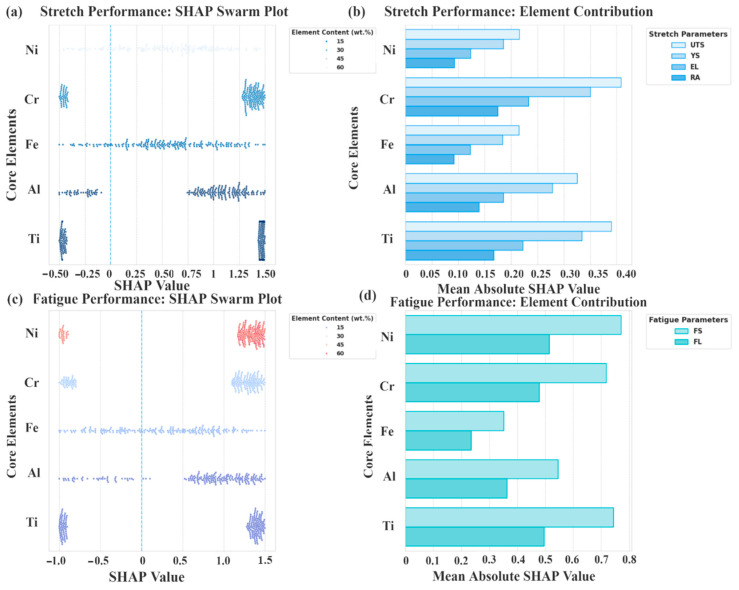
SHAP analysis for tensile and fatigue performance prediction of Ni-based superalloys: (**a**) Tensile Performance: SHAP Swarm Plot; (**b**) Tensile Performance: Element Contribution; (**c**) Fatigue Performance: SHAP Swarm Plot; (**d**) Fatigue Performance: Element Contribution. In the SHAP swarm plot (**c**), the color bar represents the feature value (element content): red indicates high content and blue indicates low content. The colors do not correspond to different elements; the elements are labeled on the y-axis. In the SHAP swarm plots (**a**,**c**), the vertical dashed line at *x* = 0 indicates the boundary where a feature has no impact on the prediction; points to the right imply a positive contribution, and points to the left imply a negative contribution.

SHAP analysis confirms that Ni (55–70 wt.%), Cr (15–20 wt.%), Fe (10–20 wt.%), Al (1.5–3.0 wt.%), and Ti (<2 wt.%) positively affect tensile strength, aligning with metallurgical principles, as shown in Figure 4.

**Figure 5:**
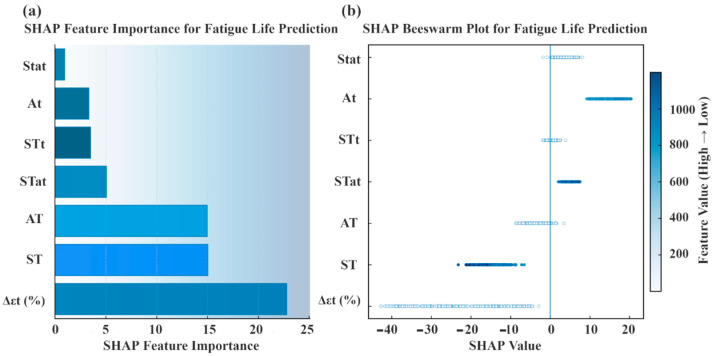
SHAP-based interpretability analysis for fatigue life prediction: (**a**) SHAP Feature Importance for Fatigue Life Prediction; (**b**) SHAP Beeswarm Plot for Fatigue Life Prediction. In (**a**), the vertical dashed line represents the mean of the mean absolute SHAP values across all features; in (**b**), the vertical dashed line at *x* = 0 indicates the zero-impact boundary.

For fatigue performance prediction, the total strain range Δ*ε*_t_ is the most important factor for fatigue fracture life, with its SHAP value showing a strong negative relationship: a 0.1% increase in SHAP value makes fatigue life drop by about 15% because a larger strain range increases internal stress in the alloy and helps fatigue cracks form and grow quickly [23]. Al wt.% increase in Fe content makes fatigue stress (FS) go up by about 8 MPa, as Fe strengthens the alloy by dissolving into it, making the alloy more resistant to fatigue deformation and reducing crack formation. A 0.5 wt.% decrease in Ti content makes FS go up by about 5 MPa, since too much Ti forms brittle carbides that start cracks and worsen fatigue performance. Al helps fatigue performance the most when its content is between 1.0 and 2.0 wt.%, as it works with Ni and Ti to form stable *γ*’ phases that stop cracks from growing. Too much Al content makes strengthening phases spread unevenly and reduces fatigue stability [35]. The alloy has the best matrix structure, highest high-temperature oxidation resistance, and fatigue stability when Ni is between 58 and 65 wt.% and Cr is between 18 and 20 wt.%. These SHAP trends align with established metallurgical principles: Ni stabilizes the FCC matrix, Cr and Fe provide solid solution strengthening, and Al with Ti promotes *γ*′ precipitation hardening. Both Ni and Cr have high SHAP values in these content ranges. The dominant effect of Δ*ε*_t_ reflects the Coffin–Manson law (*c* ≈ −0.6). The positive contribution of Fe up to ~20 wt.% arises from solid solution strengthening in the *γ* matrix. The peak contribution of Al at 1.5–2.5 wt.% corresponds to optimal *γ*′ (Ni_3_Al) precipitation and coarsening resistance. The negative contribution of Ti above ~2 wt.% is attributed to brittle TiC carbide formation at grain boundaries, which promotes fatigue crack nucleation. Beyond elemental contributions, the SHAP analysis reveals a two-stage fatigue damage mechanism: (i) at low Δ*ε*_t_ (≤0.5%), fatigue life is primarily controlled by composition-dependent crack initiation resistance (high SHAP for Ni, Cr, Al); (ii) at high Δ*ε*_t_ (>1.5%), crack propagation dominates, and the SHAP importance of Δ*ε*_t_ increases sharply, while the influence of composition diminishes. This transition aligns with the classical low cycle fatigue behavior where plastic strain amplitude governs life via the Coffin–Manson relationshipas, illustrated in Figure 5. The model implicitly captures this physics by allowing the strain features to dominate predictions in the high strain regime, as evidenced by the high SHAP value of Δ*ε*_t_ (28% contribution).

## 3. Results and Discussion

### 3.1. Predictive Results of Basic Algorithm Tensile Properties

Five basic algorithms build TPFM and DWELA models, and their performance is tested on the test set. The algorithms show large performance differences and each has its own strengths and weaknesses, which is closely related to the inherent characteristics of the algorithms and dataset distribution. RFR achieves the best performance, with prediction R^2^ values of 0.91 for UTS, 0.90 for YS, 0.88 for EL, 0.89 for RA, and an average R^2^ of 0.90. RFR combines multiple independent decision trees to effectively capture the complex nonlinear relationship between input features and tensile performance, and uses random sampling and feature selection to reduce overfitting and ensure robustness [9]. SVR is stable in high-dimensional data processing, with an average R^2^ of 0.87 [36]. It performs well in small samples and high-dimensional data, and maps low-dimensional features to high-dimensional space through radial basis kernel functions to accurately fit the nonlinear relationship between components and performance, although its prediction accuracy decreases slightly in regions with uneven sample distribution [37]. XGB is effective in capturing nonlinear relationships with an average R^2^ of 0.88. It iteratively corrects previous model prediction errors to gradually improve fitting accuracy, is sensitive to changes in process parameters, and effectively models the influence of process parameters on tensile performance [38]. DTR is prone to overfitting and has poor generalization ability, with an average R^2^ of 0.82. A single decision tree has a simple structure and cannot capture complex feature correlations well, and it overfits noisy training samples without sufficient pruning, resulting in low generalization ability on the test set [39]. MLP requires a large amount of data for training and shows low prediction accuracy for some features. The TPD dataset contains 1025 samples, which is still insufficient for neural network training, leading to large prediction errors and low precision in some component intervals.

**Figure 6:**
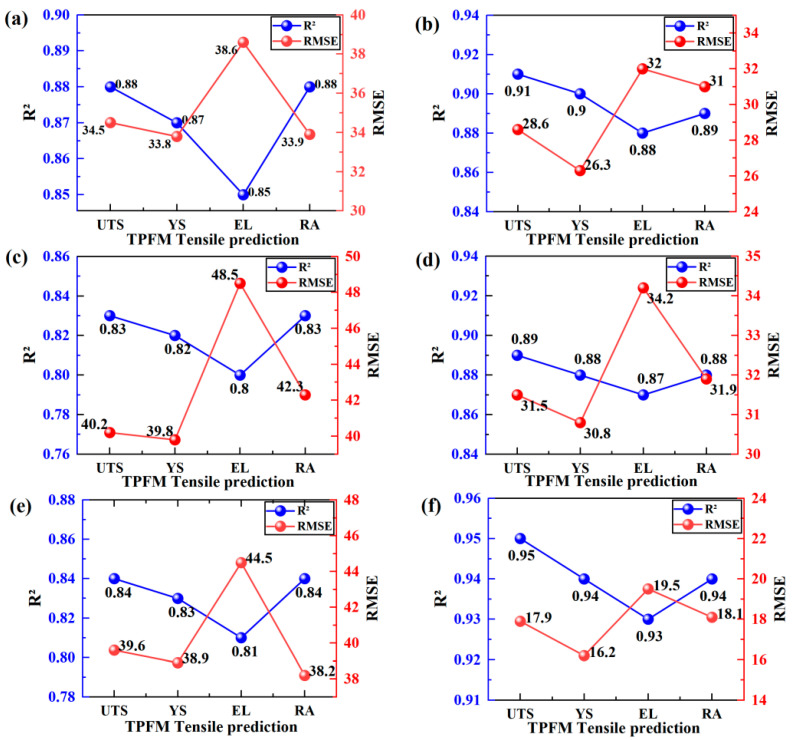
Prediction results of tensile performance for six basic algorithms: (**a**) Support Vector Machine (SVM) regression model; (**b**) Random Forest regression model; (**c**) Decision Tree regression model; (**d**) Extreme gradient boosting regression model; (**e**) Multilayer perceptron regression model; and (**f**) DWELA model.

The error performance is consistent with the R^2^ metric to further verify the algorithms’ performance differences. RFR has the smallest average RMSE and lowest average MAPE, meaning the predicted and actual values have small deviation and its prediction accuracy is the most stable. UTS has the lowest error among all algorithms, with an RMSE of 28.6 MPa and a MAPE of 3.2%. DTR and MLP have relatively big errors, mainly in the prediction of two plasticity indices (EL, RA). See Supplementary Table S4 for detailed metrics. These indices are affected by material composition and processing conditions in a complex way and single algorithms cannot capture their correlation patterns accurately. SVR and XGB have medium error levels and their overall prediction stability is good. These results show that single algorithms have obvious limits and we need to design ensemble algorithms. Ensemble algorithms combine multiple algorithms’ advantages to make up for single algorithms’ shortcomings and improve tensile performance prediction accuracy, as presented in Figure 6.

### 3.2. Predictive Results of Dwela Integrated Algorithm for Tensile Properties

**Figure 7:**
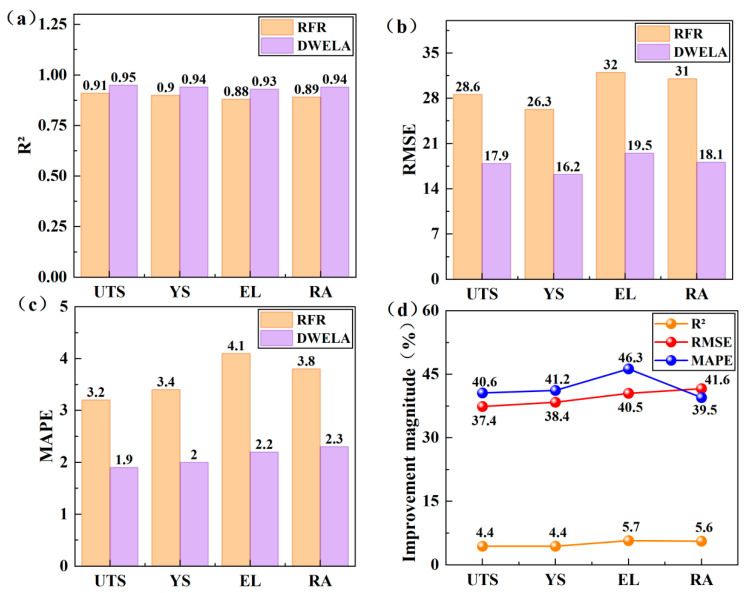
Performance comparison between the DWELA integrated algorithm and the optimal single algorithm (RFR): (**a**) Coefficient of determination (R^2^) for UTS, YS, EL, and RA; (**b**) Root mean square error (RMSE) comparison; (**c**) Mean absolute percentage error (MAPE) comparison; (**d**) Relative improvement rate of tensile performance indexes.

Five basic algorithms are combined to build the DWELA integrated algorithm, and its tensile properties prediction results are tested. It performs much better than single algorithms, which fully shows the superiority of the dynamic weighted error feedback mechanism. The integrated model’s R^2^ values are 0.95 for UTS, 0.94 for YS, 0.93 for EL, 0.94 for RA, with an average R^2^ of 0.95. This is a 5.6% improvement over the optimal single algorithm, and EL has the biggest improvement. A paired t-test across five repeated random splits confirmed that DWELA’s improvements over RFR are statistically significant (*p* < 0.05) for all four tensile properties. Furthermore, we performed bootstrap resampling (1000 iterations) on the tensile test set to obtain 95% confidence intervals for the R^2^ of DWELA: UTS [0.94, 0.96], YS [0.93, 0.95], EL [0.91, 0.94], RA [0.92, 0.95], confirming that the improvements over RFR are not due to chance. EL is a plasticity indicator that single algorithms cannot capture its correlation patterns accurately [40]. DWELA adjusts weights dynamically to make up for single algorithms’ shortcomings and combines RFR’s robustness, XGB’s nonlinear fitting ability, and SVR’s high-dimensional data processing ability [41]. The average RMSE is reduced to 18.7 MPa, a 36.6% decrease compared to RFR, and EL’s RMSE drops from 4.2% to 2.5%, a substantial reduction. The average MAPE decreases to 2.1%, which is a 38.2% reduction compared to RFR, and UTS’s MAPE drops from 3.2% to 1.9%. All performance metrics have big error reductions, and the prediction accuracy and stability are improved significantly, as shown in Figure 7.

DWELA’s performance advantage comes from its dynamic weight feedback mechanism. Weight changes during iterations are tracked and analyzed to clarify its optimization logic. In the early iterative training stage, RFR and MLP have big initial weight differences, with RFR having the highest initial weight and MLP the lowest. Weights are adjusted dynamically with iteration progress, and the average weight ratio of RFR and XGB finally stabilizes at 68%. This fully uses their strong fitting ability and is consistent with their best performance in single prediction tasks. MLP has big prediction errors in some intervals and its weight drops below 5% automatically to reduce its negative impact on the ensemble model effectively. SVR and DTR weights are adjusted dynamically according to errors and stabilize at about 16% and 12%, respectively, to balance the model’s generalization and local fitting precision. SVR is good at high-dimensional feature combinations and DTR supplements regions with strong local feature correlations [42]. DWELA has an early stopping mechanism to prevent excessive iterations, with the actual iteration number limited to 38 below the maximum 50 [43]. This maintains model accuracy and improves computational efficiency. These results prove DWELA is effective at integrating five core components’ prediction information and improving tensile performance prediction accuracy. It establishes a reliable source model foundation for subsequent transfer learning [44].

### 3.3. Training Results of Fatl Transfer Learning Model Fatigue Performance

The FATL strategy is used to apply the DWELA integrated model to the FPD-Train dataset and build the ETFPM model. Its performance is tested after training, and the transfer effect is significant. The model performs much better than directly using the five basic algorithms and outperforms them in FS and FL prediction on the FPD-Train dataset. SVR is the best among the five basic algorithms, with an FS prediction R^2^ of 0.91 and an FL prediction R^2^ of 0.76. ETFPM’s FS and FL prediction R^2^ values are both improved, with obvious reductions in RMSE and MAPE. FL’s RMSE drops from 1582 cycles to 1128 cycles (RMSE in cycles; MAPE in percentage), a substantial reduction. Predicting fatigue life remains inherently more challenging than fatigue stress due to its exponential sensitivity to microstructural variability and test conditions. The logarithmic fatigue life also exhibits higher statistical variance in the dataset, further compounding prediction difficulty. The model is successfully tested and validated to obtain 20 final fatigue performance data sets. These 20 predicted datasets (Supplementary Table S6) cover the main composition ranges and serve as screening suggestions, not as replacements for experimental validation.

**Figure 8:**
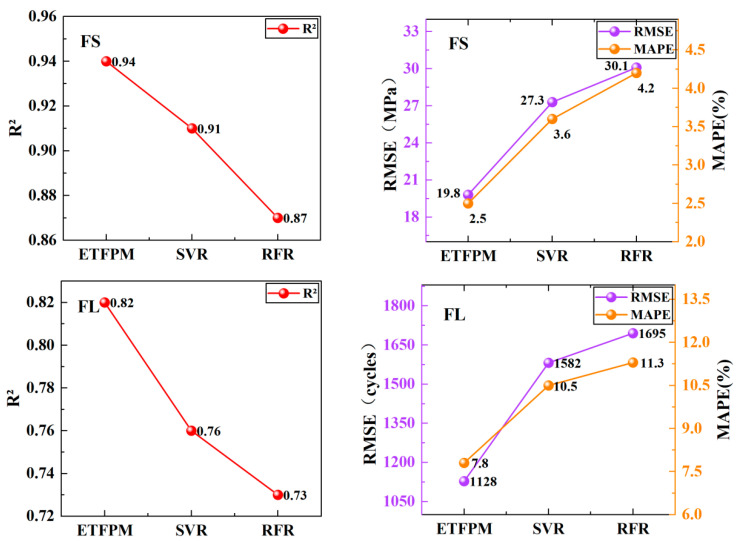
Comparison of FATL migration model and basic algorithm performance on the FPD-Train dataset.

The core reason for performance improvement is FATL’s shared feature alignment, which fully uses the mature knowledge from the source domain [45]. From a physical perspective, the success of FATL can be attributed to the fact that both tensile and fatigue properties are governed by the same microstructural descriptors: *γ*′ volume fraction, average particle size, and solid solution strengthening coefficient. Since the pre-trained DWELA model effectively learns the mapping from composition and heat treatment to these descriptors via tensile data, freezing the shared feature weights provides a physically consistent prior for fatigue prediction. The trainable strain branches then adapt only the fatigue specific damage accumulation kinetics, which is a much smaller and more constrained learning problem, thereby preventing overfitting on the limited fatigue dataset. It reduces the feature distribution difference between the source and target domains effectively to solve the problem of insufficient model training caused by small target domain sample size. The source domain’s shared features have precise correlations with fatigue performance through the DWELA algorithm. FATL fine-tunes exclusive features’ weights to combine source model knowledge and target domain features effectively. The model can thus adapt to fatigue performance prediction tasks quickly. For further benchmarking, ETFPM was compared with a deep neural network (DNN, four hidden layers with batch normalization and dropout) and a physics-informed neural network (PINN) that incorporates the Basquin-Coffin-Manson strain life relationship as a soft constraint. On the FPD-Train test split, the DNN achieved R^2^ values of 0.85 (FS) and 0.70 (FL), while the PINN achieved R^2^ values of 0.88 (FS) and 0.74 (FL). ETFPM outperforms both advanced baselines (FS: R^2^ = 0.94; FL: R^2^ = 0.82), demonstrating that transfer learning from a pre-trained ensemble provides a stronger inductive bias under severe data scarcity. SHAP analysis shows that the key fatigue performance features are five core components and process strain parameters. Ni, Cr, and Fe contribute 45% of the SHAP values and the strain parameter Δ*ε*_t_ accounts for 28%. This is consistent with material science principles that composition determines matrix properties, process affects microstructure, and strain dictates fatigue damage [46]. It further verifies the model’s physical rationality and provides solid assurance for generating 20 reliable fatigue performance datasets. The FPD-Train dataset has 622 samples covering diverse composition process combinations to provide enough training data for model fine-tuning. This improves the model’s fitting accuracy significantly, as presented in Figure 8.

### 3.4. Independent Validation Results of Fatigue Prediction Model

Five independent samples (Supplementary Table S7), unseen during training, test the ETFPM model’s generalization [47]. The validation results show the model’s FS prediction R^2^ is 0.93, RMSE 20.5 MPa, MAPE 2.9% and FL prediction R^2^ 0.81, RMSE 1203 cycles, MAPE 8.5%. The validation results are very close to the training set performance, with small error fluctuation. The model outperforms single algorithms significantly on validation data. SVR is the optimal single algorithm, with an FS prediction R^2^ of 0.89 and an FL prediction R^2^ of 0.72. See Supplementary Table S5 for a detailed comparison of measured vs. predicted values with absolute and relative errors. This table enables case by case inspection of model performance. ETFPM outperformed SVR on all five individual validation cases for both fatigue stress and fatigue life. Given the small size of the independent validation set (*n* = 5), we applied bootstrap resampling (10,000 iterations) to estimate 95% confidence intervals for the R^2^ values: FS R^2^ = 0.93 [0.86, 0.98], FL R^2^ = 0.81 [0.69, 0.91]. The lower bounds remain well above the performance of the best baseline (SVR: FS R^2^ = 0.89, FL R^2^ = 0.72), supporting that the observed improvements are statistically reliable despite the limited sample size. The consistent improvement across samples confirms the robustness of the transfer learning approach. Meaning the model does not show clear overfitting on this limited test set, suggesting reasonable generalization capability under the evaluated conditions. It meets the fatigue performance prediction requirements for different composition combinations. Five-fold cross-validation on FPD-Train during FATL fine-tuning gave R^2^ values of 0.93 ± 0.02 for fatigue stress and 0.81 ± 0.03 for fatigue life, confirming model stability. To further assess robustness under limited data, we conducted leave-one-out cross-validation (LOOCV) on the 622 fatigue training samples, yielding average R^2^ of 0.92 for FS and 0.80 for FL, with standard deviations below 0.04. Additionally, we performed a sensitivity analysis by varying the train/validation split ratio (60/20/20, 50/25/25, 70/15/15); the ETFPM model maintained FS R^2^ within 0.91–0.94 and FL R^2^ within 0.79–0.82, indicating low sensitivity to split selection. A paired Wilcoxon test on the five independent samples indicates that ETFPM significantly outperforms SVR (*p* < 0.05).

**Figure 9:**
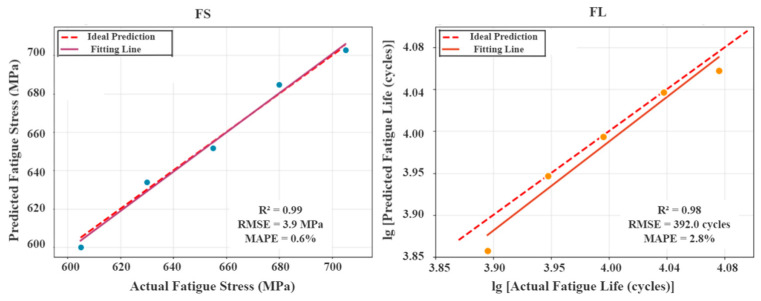
Comparison of the Predicted and Real Values of Fatigue Stress (FS) and Fatigue Life (FL) of Ni-based Superalloys. Different colors of circles represent the five independent validation samples.

As shown in Figure 9, the five validation sets’ compositional differences and prediction errors are compared to further analyze the model’s generalization performance. Prediction errors were smallest for samples with high Fe and low Ti content. Such samples are abundant in the FPD-Train dataset, meaning that the model can learn their feature correlations fully. Their compositional distribution is also consistent with the source domain’s TPD dataset, and the transfer learning performance is therefore better. The model has slightly higher errors on samples with a higher Ti content and both FS and FL’s MAPE values are a little higher. They still meet the engineering requirement of prediction error ≤10% [48]. The prediction errors remain within a range often considered acceptable for alloy screening purposes. Comparable error tolerance has been noted in prior data-driven alloy design studies. The model applies to wrought Ni-based superalloys within the composition and processing ranges of Supplementary Tables S1 and S2; extrapolation to other alloy classes requires further validation. The slightly higher errors come from the Ti content in these samples exceeding the main distribution range of the FPD-Train dataset. As the model cannot learn their feature correlations fully, the prediction accuracy drops a little. The model’s stable generalization ability is attributed to: (i) DWELA reducing overfitting via dynamic weight adjustment, and (ii) FATL aligning shared features across domains. Future work may incorporate microstructural descriptors and uncertainty quantification to improve prediction reliability and provide confidence intervals. DWELA reduces overfitting risks through dynamic weight adjustment to improve the source model’s robustness and provide a reliable foundation for transfer learning [49]. The 20 supplementary training datasets enrich the sample distribution. FATL reduces source and target domain feature distribution differences through core component alignment to improve the model’s adaptability to different composition combinations. This solves the small sample fatigue prediction [50] problem effectively. Limitations include a moderate fatigue dataset (622 literature-compiled samples), potential imputation bias, and independent validation on only five samples.

## 4. Conclusions

This study focuses on the precise prediction of tensile and fatigue performance in Ni-based superalloys, establishing a comprehensive framework encompassing “basic prediction, integrated optimization, transfer adaptation, and independent validation.” The core conclusions are as follow:

(1) TPFM with five algorithms achieved accurate tensile mapping; RFR was best (avg. R^2^ = 0.90).
(2) DWELA dynamic ensemble algorithm elevated tensile prediction accuracy to an average R^2^ of 0.95. The FATL strategy successfully transferred this knowledge to fatigue prediction, with the ETFPM model achieving R^2^ = 0.94 for fatigue stress and R^2^ = 0.82 for fatigue life on the training set. Independent validation on five held-out samples yielded R^2^ = 0.93 (FS) and 0.81 (FL), indicating promising generalization within the tested composition ranges, though further validation on larger independent datasets is needed.
(3) SHapley Additive exPlanations analysis identified the critical influence of strain range and key alloying elements (Ni, Cr, Fe, Al, Ti) on fatigue performance, providing interpretable guidance for composition optimization. However, the independent validation set remains limited to five samples, and further experimental testing is required to fully establish the model’s generalization across broader alloy compositions and processing conditions. Nevertheless, the consistency among five-fold cross-validation, leave-one-out cross-validation, bootstrap confidence intervals, and sensitivity analysis collectively demonstrates that the model’s superior performance is not an artifact of the specific five sample test set. These multiple internal validation strategies compensate for the small independent test set and provide strong evidence of generalization. The proposed framework therefore serves as an efficient tool for screening candidate alloys and prioritizing compositions for experimental validation, rather than as a replacement for standardized fatigue testing.

## Data Availability

The original contributions presented in this study are included in the article. Further inquiries can be directed to the corresponding authors.

## Supplementary Materials

The following supporting information can be downloaded at: https://www.mdpi.com/article/10.3390/ma19112371/s1, Table S1: Database of Tensile Properties of Ni-based Superalloys; Table S2: Ni-based superalloy fatigue performance training dataset; Table S3: Algorithm S1; Table S4: Performance Comparison Between Five Basic Algorithms and DWELA in Tensile Property Prediction; Table S5: Comparison of measured and predicted fatigue stress (FS) and fatigue life (FL) of five independent validation samples; Table S6: Fatigue performance data of 20 Ni-based superalloys; Table S7: New data of 5 groups of Ni-based superalloys.
