# Supplementary material for: Dynamic Ensemble Learning with Transfer Learning for Fatigue Performance Prediction in Ni-Based Superalloys
Source: Materials (Basel). 2026 Jun 2;19(11):2371. doi: 10.3390/ma19112371 (PMC13258454; doi:10.3390/ma19112371)
Supplement: Supplementary file 1 [file materials-19-02371-s001.zip › materials-4221296-supplementary.pdf]

Supplementary Table S1. Database of Tensile Properties of Ni-based Superalloys.

Type	Variable	Symbol	Minimum	Maximum
Inputs	Nickel (wt.%)	Ni	46.707	75.715
	Chromium (wt.%)	Cr	12.690	33.810
	Iron (wt.%)	Fe	0.000	20.477
	Aluminum (wt.%)	Al	0.400	3.700
	Titanium (wt.%)	Ti	0.047	5.040
	Temperature (℃)	T	19.00	1200.00
	Solid solution temperature (℃)	ST	0.00	1140.00
	Solid solution time (h)	STt	0.00	48.00
	Stabilizing aging temperature (℃)	STat	0.00	1150.00
	Stabilization aging time (h)	Stat	0.00	75.00
	Aging temperature (℃)	AT	0.00	950.00
	Aging time (h)	At	0.00	36.00
Outputs	Tensile strength (MPa)	UTS	20.00	1834.00
	Yield strength (MPa)	YS	41.00	1477.00
	Elongation (%)	EL	2.50	136.00
	Reduction of area (%)	RA	10.79	76.55

Supplementary Table S2. Ni-based superalloy fatigue performance training dataset.

Type	Variable	Symbol	Minimum	Maximum
Inputs	Nickel (wt.%)	Ni	46.707	75.715
	Chromium (wt.%)	Cr	12.690	33.810
	Iron (wt.%)	Fe	0.000	20.477
	Aluminum (wt.%)	Al	0.400	3.700
	Titanium (wt.%)	Ti	0.047	5.040
	Solid solution temperature (℃)	ST	0	1260
	Solid solution time (h)	STt	0	8
	Stabilizing aging temperature (℃)	STat	0	1120
	Stabilization aging time (h)	Stat	0	24
	Aging temperature (℃)	AT	0	980
	Aging time (h)	At	0	32
	Total strain range (%)	$\Delta\varepsilon_t$	0.00247	4.065
	Elastic strain range (%)	$\Delta\varepsilon_e$	0.00224	1.802
	Plastic strain range (%)	$\Delta\varepsilon_p$	-0.00037	3.375
	Temperature (℃)	T	20	1000
Outputs	Fatigue stress (MPa)	FS	166	1203
	Fatigue life (Cycles)	FL	62	275518

Supplementary Table S3. Algorithm S1.

**Algorithm S1: Dynamic Weighted Error Feedback Ensemble Algorithm (DWELA)**
Input:
Source domain training set D_s^{train}(size=1,025×12)
Validation set D_s^{val}(25% *of tensile data*)
Base regressors: SVR, RFR, DTR, XGB, MLP
Maximum iterations $T_{\max}$ =50,*early stopping patience p*=5
Weight bounds [0.01,0.6], convergence threshold ϵ=10^{-4}
Output: Ensemble model with optimized weights
Train each base model M_k *on* D_s^{train}
Compute validation RMSE $\mathrm{RMSE_k}$ for each M_k on D_s^{val}
Initialize weights:$w_{k,0} = \frac{1/RMSE_k}{\sum_{j=1}^{5} 1/RMSE_j}$
Set$t$=0,*best_RMSE*=∞,*counter*=0
while $t<T_{\max}$ and $counter<p$ do
$t=t+1$
Obtain predictions $\mathrm{y_{k,pred}^{(t)}}$ from each model on $\mathrm{D_s^{val}}$
Compute ensemble prediction $y_{ens}^{(t)}=\sum_{k=1}^{5} w_{k,t-1} \cdot y_{k,pred}^{(t)}$
Compute ensemble RMSE $RMSE_{ens}^{(t)}$
if $RMSE_{ens}^{(t)} < best\_RMSE - \epsilon\ then$
$best\_RMSE=RMSE_{ens}^{(t)}$,*counter*=0
else
counter=*counter*+1
end if
for k=1 *to* 5 *do*
Compute current base RMSE $RMSE_{k,t}$ *on* D_s^{val}
Update weight:$w_{k,t}=w_{k,t-1} \cdot \frac{RMSE_{k,t-1}}{RMSE_{k,t}}$
end for
Normalize weights:$w_{k,t}=w_{k,t}/\sum_{j=1}^{5} w_{j,t}$
Clip weights to [0.01,0.6] and renormalize
end while
Output final weights $w_k^*=w_{k,t}$ and the ensemble model.

Supplementary Table S4. Performance Comparison Between Five Basic Algorithms and DWELA in Tensile Property Prediction.

Model	Metric	UTS	YS	EL	RA
SVR	R^2	0.87 ± 0.02	0.86 ± 0.02	0.84 ± 0.03	0.85 ± 0.02
	RMSE (MPa / %)	32.4 ± 1.5	28.7 ± 1.2	4.1 ± 0.3	3.9 ± 0.2
	MAPE (%)	3.8 ± 0.2	3.5 ± 0.2	4.2 ± 0.3	3.9 ± 0.2
RFR	R^2	0.91 ± 0.01	0.90 ± 0.01	0.88 ± 0.02	0.89 ± 0.01
	RMSE (MPa / %)	28.6 ± 1.0	25.4 ± 0.9	3.5 ± 0.2	3.2 ± 0.2
	MAPE (%)	3.2 ± 0.1	3.0 ± 0.1	3.7 ± 0.2	3.4 ± 0.1
DTR	R^2	0.82 ± 0.03	0.81 ± 0.03	0.80 ± 0.04	0.81 ± 0.03
	RMSE (MPa / %)	38.5 ± 2.0	34.1 ± 1.8	5.2 ± 0.4	4.8 ± 0.3
	MAPE (%)	4.5 ± 0.3	4.2 ± 0.3	5.1 ± 0.4	4.6 ± 0.3
XGB	R^2	0.89 ± 0.01	0.88 ± 0.02	0.86 ± 0.02	0.88 ± 0.02
	RMSE (MPa / %)	30.2 ± 1.2	27.1 ± 1.0	3.9 ± 0.2	3.5 ± 0.2
	MAPE (%)	3.4 ± 0.1	3.2 ± 0.2	4.0 ± 0.2	3.7 ± 0.2
MLP	R^2	0.83 ± 0.03	0.82 ± 0.03	0.79 ± 0.04	0.80 ± 0.03
	RMSE (MPa / %)	37.1 ± 2.0	32.5 ± 1.5	5.5 ± 0.4	5.1 ± 0.3
	MAPE (%)	4.3 ± 0.3	4.0 ± 0.3	5.3 ± 0.4	4.8 ± 0.3
DWELA	R^2	0.95 ± 0.01	0.94 ± 0.01	0.93 ± 0.01	0.94 ± 0.01
	RMSE (MPa / %)	20.5 ± 0.8	18.2 ± 0.7	2.5 ± 0.1	2.2 ± 0.1
	MAPE (%)	1.9 ± 0.1	1.8 ± 0.1	2.1 ± 0.1	1.9 ± 0.1

Supplementary Table S5. Comparison of measured and predicted fatigue stress (FS) and fatigue life (FL) of five independent validation samples.

Sample ID	Fatigue Stress (FS)				Fatigue Life (FL)				
	Measured (MPa)	Predicted (MPa)	Absolute Error (MPa)	Relative Error (%)	Measured (cycles)	Predicted (cycles)	Absolute Error (cycles)	Relative Error (%)	Relative Error (%)
1	685	678	7	1.02	12450	13120	670	5.38	5.38
2	812	798	14	1.72	8760	8320	440	5.02	5.02
3	554	562	8	1.44	25300	26750	1450	5.73	5.73
4	476	489	13	2.73	43200	45100	1900	4.4	4.4
5	623	631	8	1.28	18700	17900	800	4.28	4.28

Supplementary Table S6. Fatigue performance data of 20 Ni-based superalloys.

Order Number	Ni(wt.%)	Cr(wt.%)	Fe(wt.%)	Al(wt.%)	Ti(wt.%)
1	58.62	19.05	4.23	1.28	1.06
2	59.47	18.43	1.12	1.42	4.89
3	56.31	15.87	0.09	2.15	1.05
4	52.45	18.31	19.26	1.29	1.34
5	53.18	18.89	13.72	0.75	1.31
6	58.34	15.67	0.08	2.61	2.61
7	51.97	18.82	19.42	0.63	3.05
8	60.55	19.08	0.47	2.11	3.31
9	60.73	18.64	1.08	2.05	3.19

Order Number	Ni(wt.%)	Cr(wt.%)	Fe(wt.%)	Al(wt.%)	Ti(wt.%)
10	62.75	13.11	0.07	3.53	1.04
11	51.34	18.65	4.58	1.48	1.09
12	57.76	19.32	0.58	1.37	5.07
13	57.98	19.02	0.53	1.51	3.05
14	53.15	18.87	18.23	0.55	1.34
15	52.69	19.07	18.44	0.64	2.79
16	57.31	15.89	0.06	2.45	3.67
17	51.34	18.65	4.58	1.48	1.06
18	60.55	19.08	0.47	2.11	4.89
19	74.23	20.37	0.09	1.03	1.05
20	56.91	15.84	0.15	2.03	1.34

Supplementary Table S7. New data of 5 groups of Ni-based superalloys.

Order Number	Ni(wt.%)	Cr(wt.%)	Fe(wt.%)	Al(wt.%)	Ti(wt.%)
1	58.43	10.38	0.05	4.12	1.59
2	75.76	20.39	0.09	1.01	2.85
3	58.75	18.84	17.73	0.61	1.05
4	54.37	19.05	19.82	0.75	1.12
5	52.39	19.34	18.85	0.56	1.17
